# Supplementary material for: Geospatial and phylogenetic clustering of acute and recent HIV infections in Lilongwe, Malawi
Source: PLOS Glob Public Health. 2025 Nov 5;5(11):e0005420. doi: 10.1371/journal.pgph.0005420 (PMC12588460; doi:10.1371/journal.pgph.0005420)
Supplement: S1 Text — (DOCX) [file pgph.0005420.s001.docx]

# SUPPLEMENTARY MATERIALS


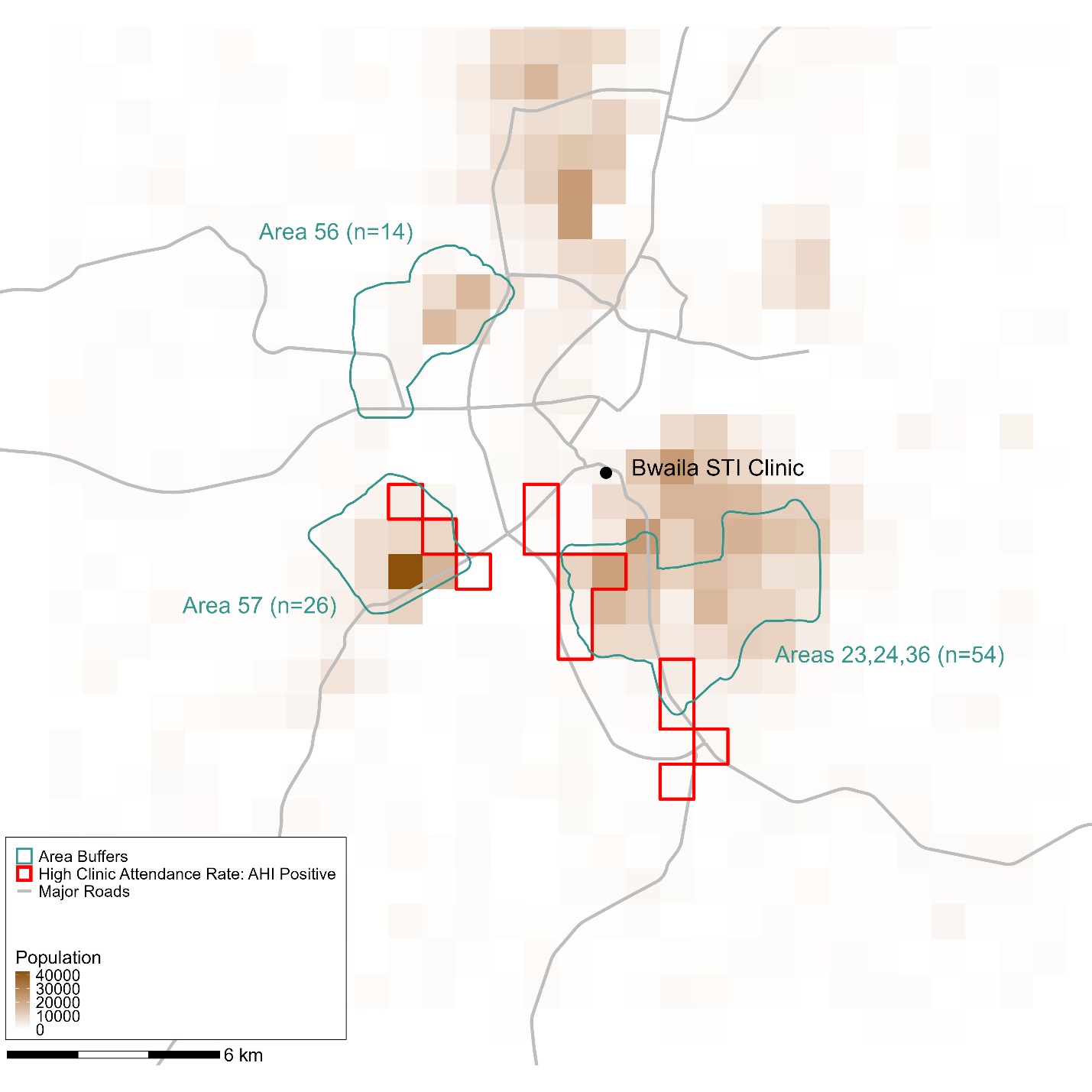


**Fig A: Sensitivity Analysis of Areas of High Clinic Attendance with AHI**

*The study area was divided into ~1x1 kilometer boxes (as opposed to ~500x500 meters in the main analysis) and high-resolution population data were aggregated and used as a denominator. The numerator was the count of clinic attendees with AHI whose residences were in the boxes. Clustering was determined using a flexible scan statistic (with α=0.1). Road shapefiles were obtained from OpenStreetMap (openstreetmap.org) under the Open Database License (ODbL) (openstreetmap.org/copyright).*

**
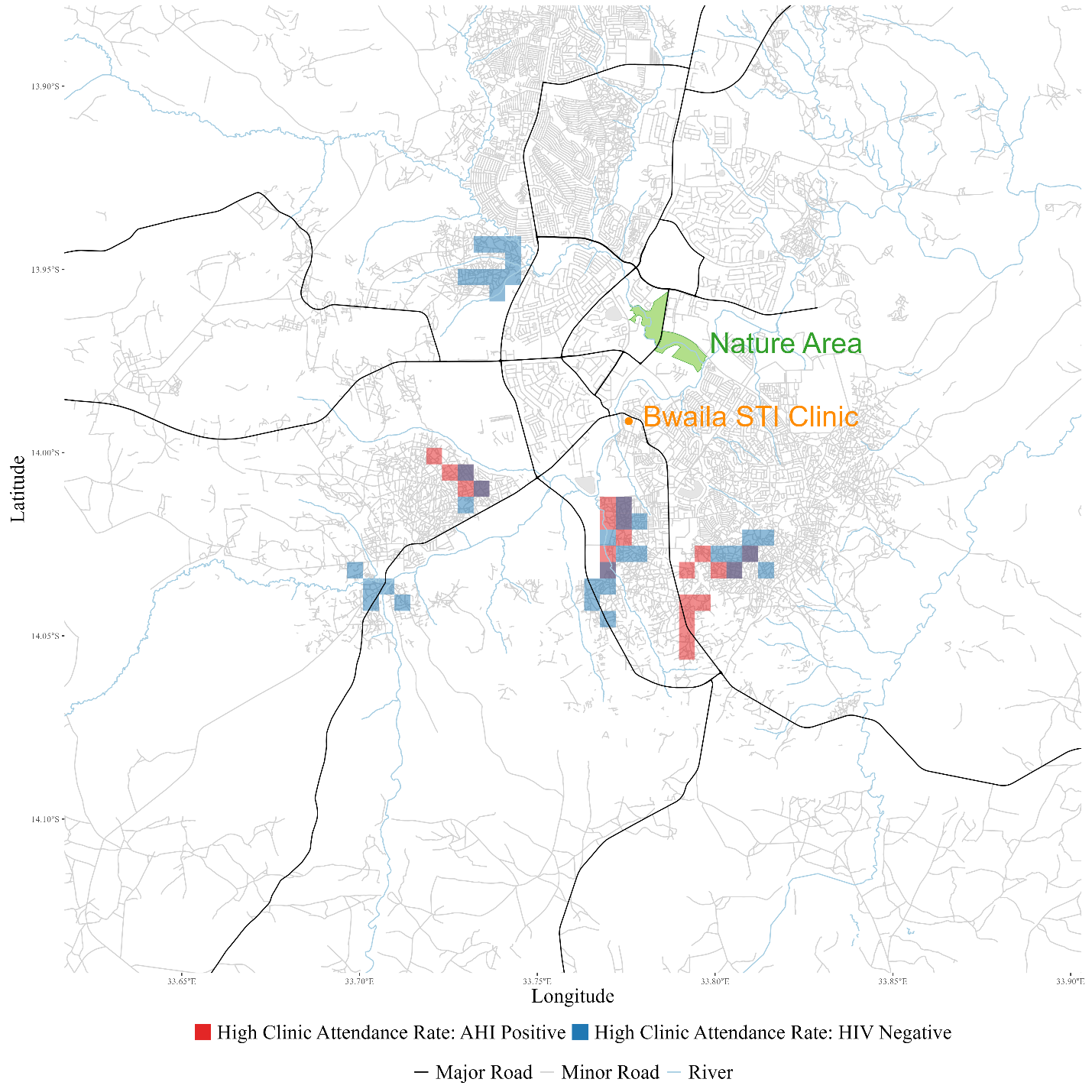
**

**Fig B: Comparison of Areas of Increased Clinic Attendance with and without AHI**

*The study area was divided into ~500x500 meter boxes and high-resolution population data were aggregated and used as a denominator. The numerator was the count of clinic attendees with AHI (red) or without HIV (blue) residing in each box. Clustering was determined using a flexible scan statistic (α=0.1). Road and river shapefiles were obtained from OpenStreetMap (openstreetmap.org) under the Open Database License (ODbL) (openstreetmap.org/copyright).*

**
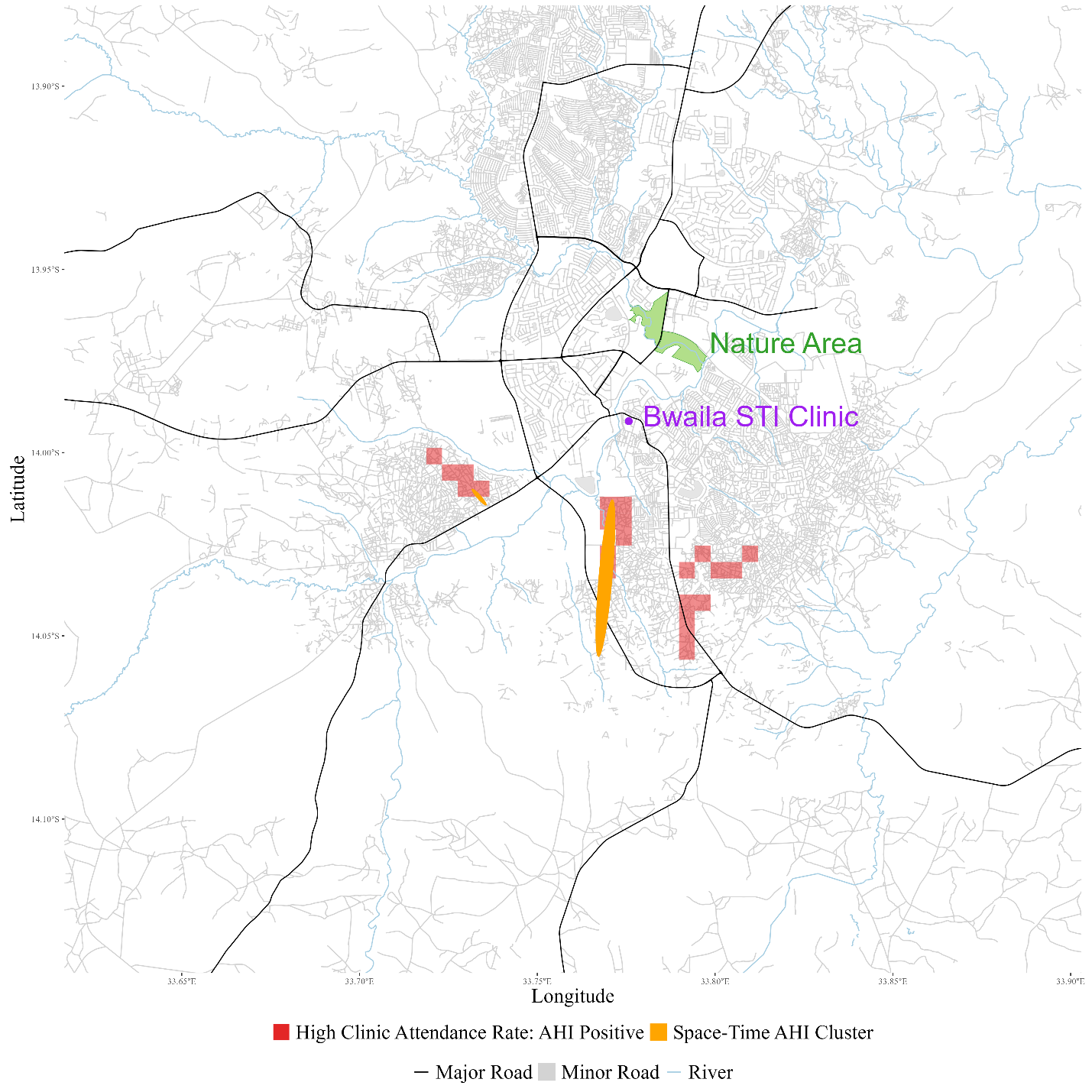
**

**Fig C: Spatiotemporal Clusters of AHI Overlayed on Purely Spatial Clusters of AHI**

*Spatiotemporal clusters were identified using a space-time permutation test (α=0.5). Cluster labels (STA1, STA2) correspond to those in Supplemental Table 1. Purely spatial clusters were determined using a flexible scan statistic (α=0.1). Road and river shapefiles were obtained from OpenStreetMap (openstreetmap.org) under the Open Database License (ODbL) (openstreetmap.org/copyright).*

**Table A: Characteristics of Spatiotemporal Clusters of Clinic Attendees with AHI**

| **Cluster ID** | **Area**  **(km^2)** | **Time Frame** | **Observed /**  **Expected AHI Count**  **(P-value)** | **Sex and Age Distribution** | **# With *gag* or *pol* Data** | **Possible Phylogenetic Linkages** |
| --- | --- | --- | --- | --- | --- | --- |
| ST Acute 1  (ST-A1) | 1.54 | 2016/11/8 to 2017/1/25 | 7 / 0.67 (0.01) | 5 men (18, 24, 23, 32, 59);  2 women (21, 25) | 4/7 | 0 |
| ST-A2 | 0.07 | 2017/6/7 to 2017/7/10 | 4 / 0.18 (0.25) | 4 women (20, 23, 28, 35) | 4/4 | 0 |

**Table B: Bayesian Information Criterion (BIC) for Evolutionary Models**

| **Region** | **Model** | **Degrees of Freedom** | **BIC** | **Selected?** |
| --- | --- | --- | --- | --- |
| gag | GTR+F | 499 | 167197.18 |  |
| gag | GTR+F+I | 500 | 151850.19 |  |
| gag | GTR+F+G4 | 500 | 141716.18 |  |
| gag | GTR+F+I+G4 | 501 | 140537.68 |  |
| gag | GTR+F+R2 | 501 | 146951.24 |  |
| gag | GTR+F+R3 | 503 | 142332.15 |  |
| gag | GTR+F+R4 | 505 | 140844.54 |  |
| gag | GTR+F+R5 | 507 | 140456.54 |  |
| gag | GTR+F+R6 | 509 | 140255.25 |  |
| gag | GTR+F+R7 | 511 | 140245.10 |  |
| gag | GTR+F+R8 | 513 | 140243.29 |  |
| gag | GTR+F+R9 | 515 | 140170.49 |  |
| gag | GTR+F+R10 | 517 | 140178.66 |  |
| gag | GTR+F+I+R2 | 502 | 143190.49 |  |
| gag | GTR+F+I+R3 | 504 | 141119.29 |  |
| gag | GTR+F+I+R4 | 506 | 140413.11 |  |
| gag | GTR+F+I+R5 | 508 | 140220.28 |  |
| gag | GTR+F+I+R6 | 510 | 140157.10 |  |
| gag | GTR+F+I+R7 | 512 | 140142.83 | Yes |
| gag | GTR+F+I+R8 | 514 | 140151.37 |  |
| gag | SYM+I+R7 | 509 | 140496.97 |  |
| gag | SYM+I+R8 | 511 | 140494.22 |  |
| gag | TVM+F+I+R7 | 511 | 140173.86 |  |
| gag | TVM+F+I+R8 | 513 | 140180.83 |  |
| gag | TVMe+I+R7 | 508 | 140696.06 |  |
| gag | TVMe+I+R8 | 510 | 140702.28 |  |
| gag | TIM3+F+I+R7 | 510 | 140386.05 |  |
| gag | TIM3+F+I+R8 | 512 | 140395.07 |  |
| gag | TIM3e+I+R7 | 507 | 141149.43 |  |
| gag | TIM3e+I+R8 | 509 | 141148.12 |  |
| gag | TIM2+F+I+R7 | 510 | 140365.37 |  |
| gag | TIM2+F+I+R8 | 512 | 140373.57 |  |
| gag | TIM2e+I+R7 | 507 | 140737.97 |  |
| gag | TIM2e+I+R8 | 509 | 140750.41 |  |
| gag | TIM+F+I+R7 | 510 | 140365.25 |  |
| gag | TIM+F+I+R8 | 512 | 140374.89 |  |
| gag | TIMe+I+R7 | 507 | 141111.67 |  |
| gag | TIMe+I+R8 | 509 | 141109.19 |  |
| gag | TPM3u+F+I+R7 | 509 | 140413.84 |  |
| gag | TPM3u+F+I+R8 | 511 | 140421.58 |  |
| gag | TPM3+I+R7 | 506 | 141311.57 |  |
| gag | TPM3+I+R8 | 508 | 141319.37 |  |
| gag | TPM2u+F+I+R7 | 509 | 140394.14 |  |
| gag | TPM2u+F+I+R8 | 511 | 140399.22 |  |
| gag | TPM2+I+R7 | 506 | 140925.76 |  |
| gag | TPM2+I+R8 | 508 | 140993.64 |  |
| gag | K3Pu+F+I+R7 | 509 | 140382.50 |  |
| gag | K3Pu+F+I+R8 | 511 | 140388.45 |  |
| gag | K3P+I+R7 | 506 | 141279.49 |  |
| gag | K3P+I+R8 | 508 | 141286.93 |  |
| gag | TN+F+I+R7 | 509 | 140543.07 |  |
| gag | TN+F+I+R8 | 511 | 140551.47 |  |
| gag | TNe+I+R7 | 506 | 141308.60 |  |
| gag | TNe+I+R8 | 508 | 141307.21 |  |
| gag | HKY+F+I+R7 | 508 | 140568.78 |  |
| gag | HKY+F+I+R8 | 510 | 140582.16 |  |
| gag | K2P+I+R7 | 505 | 141478.09 |  |
| gag | K2P+I+R8 | 507 | 141484.94 |  |
| gag | F81+F+I+R7 | 507 | 149445.67 |  |
| gag | F81+F+I+R8 | 509 | 149455.61 |  |
| gag | JC+I+R7 | 504 | 150929.29 |  |
| pol | GTR+F | 501 | 271333.01 |  |
| pol | GTR+F+I | 502 | 242757.33 |  |
| pol | GTR+F+G4 | 502 | 225943.33 |  |
| pol | GTR+F+I+G4 | 503 | 223748.32 |  |
| pol | GTR+F+R2 | 503 | 233332.05 |  |
| pol | GTR+F+R3 | 505 | 226378.80 |  |
| pol | GTR+F+R4 | 507 | 224169.84 |  |
| pol | GTR+F+R5 | 509 | 223342.43 |  |
| pol | GTR+F+R6 | 511 | 223090.22 |  |
| pol | GTR+F+R7 | 513 | 223035.92 |  |
| pol | GTR+F+R8 | 515 | 223035.10 |  |
| pol | GTR+F+R9 | 517 | 223041.39 |  |
| pol | GTR+F+I+R2 | 504 | 227702.12 |  |
| pol | GTR+F+I+R3 | 506 | 224332.65 |  |
| pol | GTR+F+I+R4 | 508 | 223400.63 |  |
| pol | GTR+F+I+R5 | 510 | 223100.63 |  |
| pol | GTR+F+I+R6 | 512 | 222988.61 |  |
| pol | GTR+F+I+R7 | 514 | 222982.04 | Yes |
| pol | GTR+F+I+R8 | 516 | 222992.16 |  |
| pol | SYM+I+R6 | 509 | 223769.57 |  |
| pol | SYM+I+R7 | 511 | 223734.41 |  |
| pol | TVM+F+I+R6 | 511 | 223167.54 |  |
| pol | TVM+F+I+R7 | 513 | 223082.07 |  |
| pol | TVMe+I+R6 | 508 | 223996.61 |  |
| pol | TVMe+I+R7 | 510 | 223957.29 |  |
| pol | TIM3+F+I+R6 | 510 | 223447.18 |  |
| pol | TIM3+F+I+R7 | 512 | 223443.59 |  |
| pol | TIM3e+I+R6 | 507 | 225038.28 |  |
| pol | TIM3e+I+R7 | 509 | 224994.25 |  |
| pol | TIM2+F+I+R6 | 510 | 223339.88 |  |
| pol | TIM2+F+I+R7 | 512 | 223328.43 |  |
| pol | TIM2e+I+R6 | 507 | 223957.06 |  |
| pol | TIM2e+I+R7 | 509 | 223909.48 |  |
| pol | TIM+F+I+R6 | 510 | 223367.16 |  |
| pol | TIM+F+I+R7 | 512 | 223360.27 |  |
| pol | TIMe+I+R6 | 507 | 224868.64 |  |
| pol | TIMe+I+R7 | 509 | 224826.28 |  |
| pol | TPM3u+F+I+R6 | 509 | 223630.73 |  |
| pol | TPM3u+F+I+R7 | 511 | 223526.68 |  |
| pol | TPM3+I+R6 | 506 | 225198.25 |  |
| pol | TPM3+I+R7 | 508 | 225157.17 |  |
| pol | TPM2u+F+I+R6 | 509 | 223498.78 |  |
| pol | TPM2u+F+I+R7 | 511 | 223437.40 |  |
| pol | TPM2+I+R6 | 506 | 224190.61 |  |
| pol | TPM2+I+R7 | 508 | 224143.21 |  |
| pol | K3Pu+F+I+R6 | 509 | 223542.50 |  |
| pol | K3Pu+F+I+R7 | 511 | 223457.93 |  |
| pol | K3P+I+R6 | 506 | 225028.58 |  |
| pol | K3P+I+R7 | 508 | 224992.19 |  |
| pol | TN+F+I+R6 | 509 | 223647.19 |  |
| pol | TN+F+I+R7 | 511 | 223639.63 |  |
| pol | TNe+I+R6 | 506 | 225080.70 |  |
| pol | TNe+I+R7 | 508 | 225031.90 |  |
| pol | HKY+F+I+R6 | 508 | 223812.74 |  |
| pol | HKY+F+I+R7 | 510 | 223727.76 |  |
| pol | K2P+I+R6 | 505 | 225240.87 |  |
| pol | K2P+I+R7 | 507 | 225197.17 |  |
| pol | F81+F+I+R6 | 507 | 243807.83 |  |
| pol | F81+F+I+R7 | 509 | 243789.46 |  |
| pol | JC+I+R6 | 504 | 246155.96 |  |
| pol | JC+I+R7 | 506 | 246141.81 |  |
