# Supplementary material for: Geospatial and phylogenetic clustering of acute and recent HIV infections in Lilongwe, Malawi
Source: PLOS Glob Public Health. 2025 Nov 5;5(11):e0005420. doi: 10.1371/journal.pgph.0005420 (PMC12588460; doi:10.1371/journal.pgph.0005420)
Supplement: Attachment — Submitted filename: PGPH-D-25-01598-Review.docx [file pgph.0005420.s002.docx]

**Title: Geospatial and Phylogenetic Clustering of Acute and Recent HIV Infections in Lilongwe, Malawi**

**Overall Assesment**

This manuscript tackles a highly relevant public health challenge detecting and characterizing clustering of acute and recent HIV infections (AHI/RHI) in Malawi using a combination of geospatial statistics and phylogenetic analysis. The dual approach is innovative and contributes significantly to understanding early HIV transmission dynamics. The findings have implications for designing targeted HIV prevention strategies, especially in urban African settings where generalized epidemics coexist with concentrated sub-epidemics.

The manuscript is well-motivated and methodologically rich, but there are areas where clarity, integration of findings, and framing of public health implications can be strengthened.

**Major concerns**

1. 2019 dataset is quite old, a more recent one would be more informative given the evolving nature of the epidemic.
2. While the spatial and the phylogenetic analysis are rigorous, they appear to operate in parallel. The key result is no phylogenetic clusters overlapped with geospatial clusters. This is important but under-discussed. The discussion should explore why this might be the case for example high partner mobility, clinic referral bias, or the possibility that transmission occurs in social/sexual venues rather than within neighborhoods.
3. Methodology needs to be expounded, even if it is under supplemetary materials: Evolutionary models; what are they, how many were they, what was the BIC values for the models, what is IQTree and so on.
4. AHI vs. RHI definitions are clear, but the distinction between acute, recent, and chronic infections could be summarized more succinctly in the results/discussion to aid readability for non-specialist readers.
5. The choice of 500×500m grids is justified, but results from the 1×1 km sensitivity analysis should be more explicitly reported in the results. Were clusters robust to this change?
6. The use of <2% distance for gag/pol and <4.5% for whole genome is appropriate, but the rationale for mixing these thresholds could be clarified. Why not apply a single genome-wide criterion?
7. The decision to allow looser clustering when gag/pol overlap <50% needs strongerjustification.
8. The authors conclude that AHI detection at STI clinics could guide interventions. This is valid, but could be strengthened by linking to life-course approaches and combination prevention strategies (e.g., community testing, PrEP, contact tracing).
9. More explicit discussion on feasibility in Malawi is warranted: would STI clinics realistically serve as sentinel sites given health system capacity constraints?

**Minor concerns**

1. The abstract states “three phylogenetic pairs consisted of only acute/recent members” percentages or context (e.g., proportion of all clusters) would help.
2. Figure/table clarity: ensure spatial maps are legible with population density shading and case locations.
3. Avoid overuse of acronyms (AHI, RHI, CHI, PLACE, etc.) define clearly at first use in abstract and main text.
