# Supplementary material for: Geospatial and phylogenetic clustering of acute and recent HIV infections in Lilongwe, Malawi
Source: PLOS Glob Public Health. 2025 Nov 5;5(11):e0005420. doi: 10.1371/journal.pgph.0005420 (PMC12588460; doi:10.1371/journal.pgph.0005420)
Supplement: Attachment — Submitted filename: response_to_reviewers_v3.docx [file pgph.0005420.s004.docx]

**RESPONSE TO REVIEWERS**

We would like to sincerely thank the reviewers for their helpful comments and constructive critiques. Their contribution is much appreciated and has improved the manuscript. We hope that we have adequately addressed their concerns below.

**Reviewer #1: The authors present a well-written summary of largely null findings. I feel that the methodology is appropriate and the conclusions are well supported by the data. I have a few minor comments.**

**1. The major limitation of this study is the low sequencing coverage. The authors do sequence ~80% of participants with AHI, but only 25% of enrolled participants. The authors mention that they may have insufficiently sampled the sexual network by only focusing on STI clinic attendees; they should also discuss how the low sequencing coverage may impact their results.**

We have modified the text in the fourth paragraph of the discussion to better acknowledge the issue of incomplete screening coverage:

“We also note that although we sequenced the majority of AHI/RHI participants and a randomly selected subset of CHI patients, our sampling design and sequencing priorities favored referred (vs. unreferred) sexual partners, and it is possible that links to unsequenced partners were missed or that transmissions occurring during AHI presented at the clinic as chronic-chronic pairs after the early stages had passed.”

We have also added text to the immediately prior sentence to ensure that readers understand that the intent of our study was not to quantify the contribution of transmission during AHI to overall HIV incidence in Lilongwe (a pursuit that would have required even further discussion of the biases arising from the narrow slice of the transmission network we observed):

“...our study was not designed to quantify the contribution of transmission during AHI to overall HIV incidence in Lilongwe.”

**2. The authors describe venues (bars, brothels, etc.) and the proximity of participant home locations to areas with these types of venues. It's not clear to the reader how we should interpret this. Is there any data (other than proximity) linking participants to these venues? For example, did any index cases report working at or visiting these specific areas?**

Unfortunately, we did not collect any data linking participants to venues, so we can only discuss proximity and linkage by major roads to high-venue areas. We added one sentence to the second-to-last paragraph of the discussion to address this limitation and suggest the need for future research:

“We did not collect information on venue attendance from participants and thus could not conduct a more detailed investigation into the role of venues in HIV transmission, but our results suggest that future research probing links between AHI and venue attendance could be informative for intervention design.”

**3. The authors use genetic distance to infer likely recent transmission. This is not inappropriate, but I wonder if they considered model-based approaches to infer transmission events (perhaps based on the timing of diagnosis and the pattern of site mutations)? Such an approach might reveal the presence and number of unobserved intermediates between more distantly related pairs.**

The reviewer raises an excellent point that we discussed at length during the analysis phase of our study. We had considered using deep sequencing methods to support further inferences (time since transmission, direction of transmission) as suggested by the reviewer, but we concluded that simple genetic distance measures between consensus sequences would enable us to answer our primary research questions without incurring additional delays beyond those that had already arisen from laboratory disruptions caused by the COVID-19 pandemic.

**4. The authors assert in the discussion (4th paragraph) that individuals may be less likely to refer very recent sexual partners. Why do the authors think that? My expectation is that people would be more likely to refer very recent partners (recency bias).**

We had raised this possibility as a potential way in which transmission links during AHI may have been underestimated, but we realize now that this suggestion may be confusing and have thus removed this part of the sentence.

**5. Circular phylogenies are hard to read; it is difficult to compare branch lengths when they aren't plotted on the same horizontal axis. Please change these to rectangular trees and include a scale bar (subs/site) to improve interpretation.**

Thank you, we have made this change.

**Reviewer #2: Title: Geospatial and Phylogenetic Clustering of Acute and Recent HIV Infections in Lilongwe, Malawi**

**Overall Assesment**

**This manuscript tackles a highly relevant public health challenge detecting and characterizing clustering of acute and recent HIV infections (AHI/RHI) in Malawi using a combination of geospatial statistics and phylogenetic analysis. The dual approach is innovative and contributes significantly to understanding early HIV transmission dynamics. The findings have implications for designing targeted HIV prevention strategies, especially in urban African settings where generalized epidemics coexist with concentrated sub-epidemics.**

**The manuscript is well-motivated and methodologically rich, but there are areas where clarity, integration of findings, and framing of public health implications can be strengthened.**

**Major concerns**

**1. 2019 dataset is quite old, a more recent one would be more informative given the evolving nature of the epidemic.**

While a more recent dataset would be optimal, such a dataset does not exist and is difficult to envision in the time since our data were collected, given the disruptions caused (first) by the COVID-19 pandemic and (more recently) by sudden disinvestments in important global health research and funding mechanisms. In the vacuum created by these disruptions, we believe that it is crucial to leverage our unique dataset (spatial and phylogenetic data, large study of people with AHI in Africa, venue information) to draw insights for public health practice.

**2. While the spatial and the phylogenetic analysis are rigorous, they appear to operate in parallel. The key result is no phylogenetic clusters overlapped with geospatial clusters. This is important but under-discussed. The discussion should explore why this might be the case for example high partner mobility, clinic referral bias, or the possibility that transmission occurs in social/sexual venues rather than within neighborhoods.**

Thank you for this point. Your observation that our spatial and phylogenetic analyses are largely conducted in parallel is correct. Because there was no overlap between spatial and phylogenetic clusters, there were limited conclusions that we were able to draw in a phylogeographic analysis. Though we previously raised two potential explanations for the lack of overlap in the second-to-last paragraph of the Discussion section, we did not previously address the suggestion of high partner mobility. We have now added two sentences to that paragraph to raise that potential explanation: “Relatedly, transmission partners may have been unobserved due to high mobility or migration between regions outside the study area. Studies from Uganda, which find that migrants (55) and female partners of migrants (56) are at increased risk of HIV, support this possibility.”

We have also added some text in this same paragraph to more clearly link the other two possibilities we had previously raised to those mentioned above by the reviewer (clinic referral bias, the possibility of transmission at venues rather than residential neighborhoods):

“One explanation is that we insufficiently sampled the sexual networks in Lilongwe by only including STI clinic attendees and their referred partners; there were likely additional people with AHI, who were not referred to the clinic, who reside in those neighborhoods and they may have been part of a local transmission chain.”

and

“A third possible explanation is that household locations may differ meaningfully from HIV transmission locations (e.g. bars, brothels, and other venues), and that transmission partners may reside in different areas of the city. The observed connectivity among AHI geospatial clusters, venues, and major roads is compatible with this possibility.”

**3. Methodology needs to be expounded, even if it is under supplemetary materials: Evolutionary models; what are they, how many were they, what was the BIC values for the models, what is IQTree and so on.**

Thank you for this suggestion. We agree and have added all the evolutionary models tested and their respective BICs in Supplemental Table 2. IQTree is cited in the main text.

**4. AHI vs. RHI definitions are clear, but the distinction between acute, recent, and chronic infections could be summarized more succinctly in the results/discussion to aid readability for non-specialist readers.**

We define all three infection stages (acute, recent, and chronic HIV) in the Methods section and have limited the Results and Discussion sections to describing “the results of the experiments, the interpretation of these results, and the conclusions that can be drawn” in keeping with journal submission guidelines. We believe it would be cumbersome to repeat the full definitions in the results/discussion but will defer to the editor if such an addition is deemed important by the journal. We also note that we have added the word “and Chronic” to the Methods subheading above the paragraph where we introduce the definitions to more clearly signpost the availability of all three definitions.

**5. The choice of 500×500m grids is justified, but results from the 1×1 km sensitivity analysis should be more explicitly reported in the results. Were clusters robust to this change?**

We have expanded our reporting here (first paragraph of the Results section): “Our sensitivity analysis using ~1x1 km^2^ units mostly identified similar areas with elevated proportions of the population attending Bwaila STI clinic with AHI (Supplemental Figure 1): clusters A1, A2, and A4 were moderately expanded while A3 was absent. Clusters identified in this sensitivity analysis were less spatially efficient for the presence of AHI than were those in the main analysis, capturing 36/135 (27%) AHI cases in 1.5% of the populated clinic catchment area and 4.1% of the population.”

**6. The use of <2% distance for gag/pol and <4.5% for whole genome is appropriate, but the rationale for mixing these thresholds could be clarified. Why not apply a single genome-wide criterion?**

Our clustering thresholds were generally meant to be robust to missing sequence data and recombination. We have added a sentence to the “Phylogenetic Analyses” sub-section of the Methods section to explain: “These combined region-specific and whole-genome criteria allowed us to detect potential transmission pairs while remaining robust in the presence of recombination and any partial sequencing failures.”

We will note that applying only the 4.5% whole genome threshold, regardless of missingness, results in the same 14 pairs as the mixed thresholds.

**7. The decision to allow looser clustering when gag/pol overlap <50% needs strongerjustification.**

When gag and pol are absent, what is left is predominantly higher-mutation regions. We have added a sentence to the “Phylogenetic Analyses” sub-section of the Methods: “Higher thresholds when gag and pol are not present reflect increased mutation rates in other areas of the genome.”

**8. The authors conclude that AHI detection at STI clinics could guide interventions. This is valid, but could be strengthened by linking to life-course approaches and combination prevention strategies (e.g., community testing, PrEP, contact tracing).**

We have added a sentence to our conclusion: “The identification of these neighborhoods and networks may also enable the distribution of pre-exposure prophylaxis, HIV self-testing interventions, and combination prevention packages to those at high risk of infection or onward transmission.”

**9. More explicit discussion on feasibility in Malawi is warranted: would STI clinics realistically serve as sentinel sites given health system capacity constraints?**

There is a long history of prior work in Lilongwe establishing the feasibility and efficiency of AHI detection programs in STI clinic settings. We have added a sentence to the first paragraph of the Discussion to address this point:

“This type of setting has been an efficient location for AHI detection in Lilongwe for more than two decades, with numerous studies demonstrating the feasibility of such AHI detection programs and the potential promise of leveraging sociosexual networks to extend HIV prevention interventions beyond the clinic population (6,25,27,41–45).”

**Minor concerns**

**1. The abstract states “three phylogenetic pairs consisted of only acute/recent members” percentages or context (e.g., proportion of all clusters) would help.**

We have added “of the fourteen” to this sentence (“three phylogenetic pairs of the fourteen...”).

**2. Figure/table clarity: ensure spatial maps are legible with population density shading and case locations.**

We believe that the maps are legible in their current form, but we would be amenable to considering more specific suggestions. We are unable to display case household locations due to ethical concerns around publishing identifiable information.

**3. Avoid overuse of acronyms (AHI, RHI, CHI, PLACE, etc.) define clearly at first use in abstract and main text.**

For acronyms (e.g., AHI) that we use extensively throughout the manuscript, we have ensured that they are clearly defined at first instance in the abstract and main text. Several acronyms that we used less extensively have now been removed, including Female (F), Male (M), Traditional authority (TA), and Bayesian information criterion (BIC).
